# Supplementary material for: Anti-IgG Doped Melanin Nanoparticles Functionalized Quartz Tuning Fork Immunosensors for Immunoglobulin G Detection: In Vitro and In Silico Study
Source: Sensors (Basel). 2024 Jul 3;24(13):4319. doi: 10.3390/s24134319 (PMC11243786; doi:10.3390/s24134319)
Supplement: Supplementary file 1 [file sensors-24-04319-s001.zip › sensors-2932198-supplementary.pdf]

Supplementary Material

Anti-IgG doped melanin nanoparticle functionalized quartz tuning fork immunosensors for immunoglobulin G detection: *In vitro* and *in silico* study

Dilhan Tanrılı[a], Engin Baysoy[b*], Gizem Kaleli-Can[a*]

[a] Department of Biomedical Engineering, İzmir Democracy University, İzmir 35140, Türkiye

[b] Department of Biomedical Engineering, Bahçeşehir University, İstanbul, 34353, Türkiye

*Corresponding Authors: Gizem KALELİ-CAN, Engin BAYSOY

S.1. Frequency Measurements

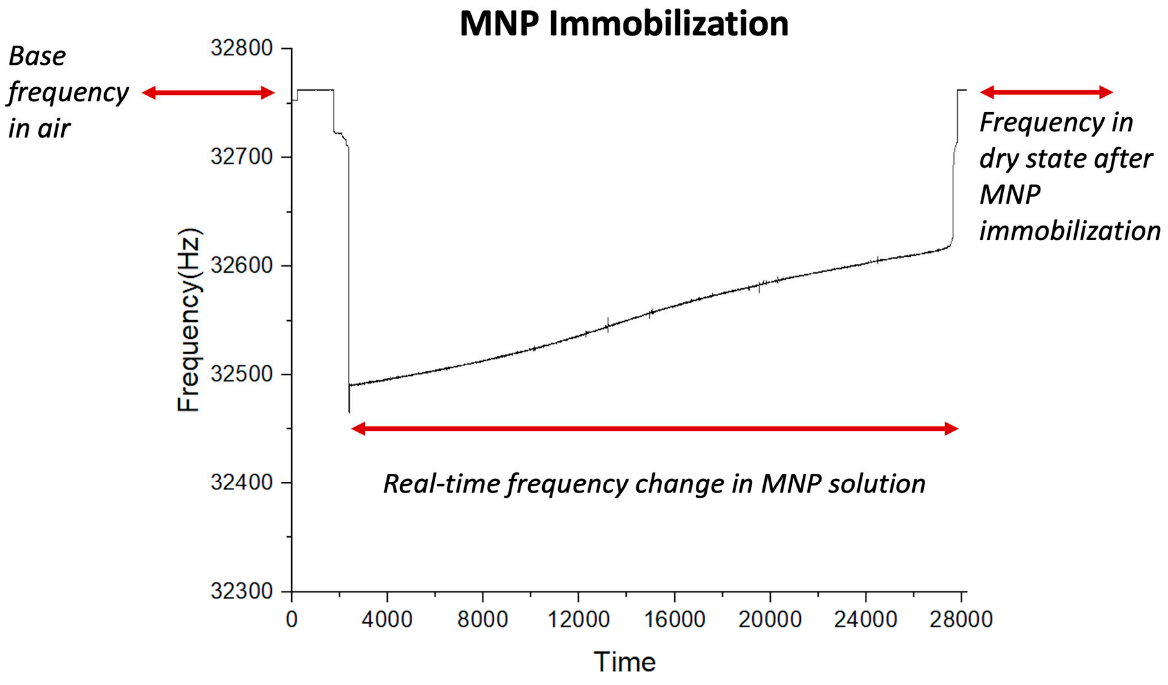

Figure S1. Real-time frequency shifts during MNP immobilization in both dry and solution.

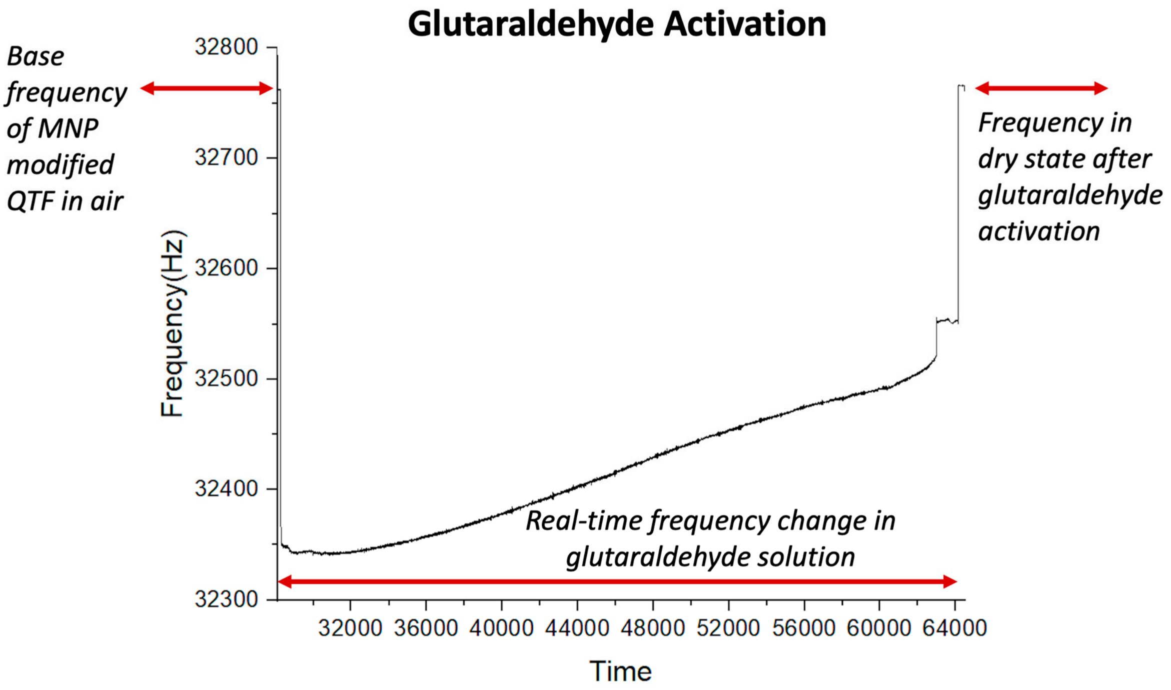

Figure S2. Real-time frequency shifts during glutaraldehyde activation in both dry and solution.

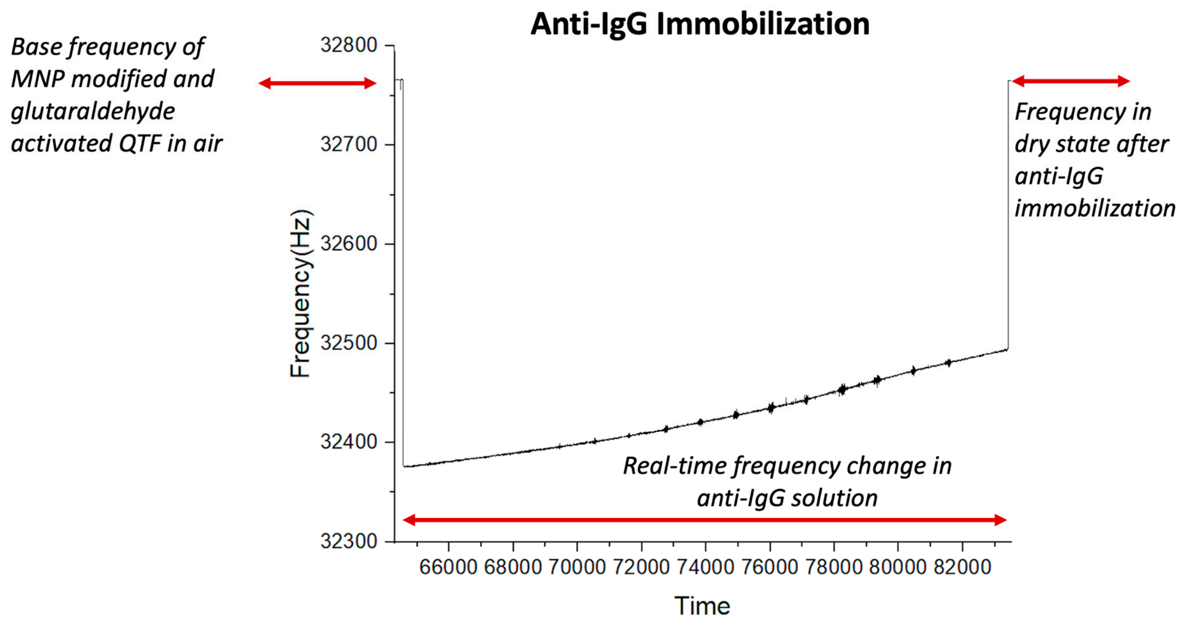

Figure S3. Real-time frequency shifts during anti-IgG immobilization in both dry and solution.

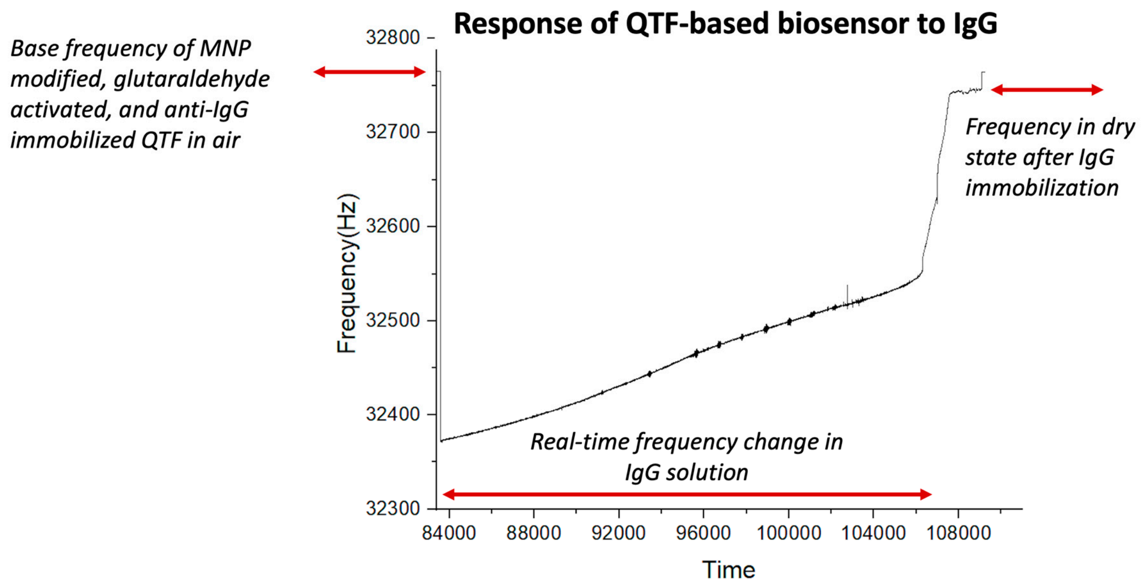

Figure S4. Real-time response to IgG in both dry and solution.

Table S1. Properties of piezoelectric crystal (Quartz LH-1978 IEEE).

Property	Value	Unit
Density	2650	kg/m3
Elasticity matrix, Voigt notation	8.67362	Pa
Coupling matrix, Voigt notation	-0.1710	C/m2
Relative permittivity	4.5	1
Young’s modulus	78x109	Pa
Poisson’s ratio	0.17	1
Electrical conductivity	1x10-18	S/m

Table S2. Geometric features of QTF model

Features	Value
Ambient Temperature (T)	293.15 [K]
Ambient Pressure (P)	1 [atm]
Applied Voltage (V0)	0.1 [V]
QTF’s Prongs Width (L1)	0.35 [mm]
QTF’s Prongs Height (L2)	10 [mm]
QTF’s Prongs Thickness (L3)	0.60 [mm]
QTF’s Body Width (G1)	1.80 [mm]
QTF’s Body Height (G2)	8.20 [mm]

Table S3. Material properties of MNP, GLU, anti-IgG, and IgG containing thin film defined in COMSOL Multiphysics.

MNP's properties			
Density	rho	1650	kg/m3
Poisson's ratio	nu	0.35	1
Young's modulus	E	2x109	Pa
Relative permittivity	epsilon	2.72	1
Electrical conductivity	Sigma	1x10-11	S/m
Mean molar mass	Mn	3183x10-4	kg/mol
GLU's properties			
Density	rho	1060	kg/m3
Relative permittivity	epsilon	23	1
Mean molar mass	Mn	100.117	kg/mol
Dynamic viscosity	mu	3.4x10-3	Pa.s
anti-IgG's properties			
Young's modulus	E	1.7x109	Pa
Poisson's ratio	Nu	0.3	1
Relative permittivity	Epsilon	10	1
Electrical conductivity	Sigma	0.000000825	S/m
Mean molar mass	Mn	14	kg/mol
IgG's properties			
Electrical conductivity	Sigma	0.000000825	S/m
Young's modulus	E	5.2 [GPa]	Pa
Poisson's ratio	Nu	0.25	1
Relative permittivity	Epsilon	10	1
Mean molar mass	Mn	150	kg/mol
